# Reduced Vitellogenesis and Female Fertility in Gper Knockout Zebrafish

**DOI:** 10.3389/fendo.2021.637691

**Published:** 2021-03-09

**Authors:** Xin-Jun Wu, Marcus Jermaul Williams, Kimberly Ann Kew, Aubrey Converse, Peter Thomas, Yong Zhu

**Affiliations:** ^1^Department of Biology, East Carolina University, Greenville, NC, United States; ^2^Department of Biochemistry and Molecular Biology, Brody School of Medicine, East Carolina University, Greenville, NC, United States; ^3^Marine Science Institute, University of Texas at Austin, Port Aransas, TX, United States

**Keywords:** G-protein coupled estrogen receptor (Gper), epidermal growth factor receptor (Egfr), oocyte growth, reduced fertility, vitellogenin

## Abstract

The role G-protein coupled estrogen receptor (GPER) plays in vertebrate reproduction remains controversial. To investigate GPER’s reproductive role, we generated a gper zebrafish mutant line (*gper^−/−^*) using TALENs. Gper mutant females exhibited reduced fertility with a 40.85% decrease in embryo production which was associated with a significant decrease in the number of Stage V (730–750 μm) ovulated oocytes. Correspondingly, the number of early vitellogenic follicles (Stage III, 400–450 µm) in *gper^−/−^* ovaries was greater than that in wildtypes (*wt*), suggesting that subsequent follicle development was retarded in the *gper^−/−^* fish. Moreover, plasma vitellogenin levels were decreased in *gper^−/−^* females, and epidermal growth factor receptor (Egfr) expression was lower in Stage III vitellogenic oocytes than in *wt* counterparts. However, hepatic nuclear estrogen receptor levels were not altered, and estrogen levels were elevated in ovarian follicles. These results suggest that Gper is involved in the control of ovarian follicle development *via* regulation of vitellogenesis and Egfr expression in zebrafish.

## Introduction

17*β*-estradiol, the predominant female sex hormone, plays a critical role in female reproductive development and function (1–3). Traditionally, 17*β*-estradiol was widely considered to act solely through intracellular receptors, nuclear estrogen receptors, facilitating their dimerization and binding to estrogen response elements in the promoters of target genes, resulting in transcription and translation. However, there is now abundant evidence that 17*β*-estradiol and other steroid hormones can also act through receptors on the cell surface to rapidly activate intracellular signaling pathways resulting in cellular responses that are often nongenomic (4). For example, 17*β*-estradiol activates a cell membrane-located G-protein coupled estrogen receptor (GPER, formally known as GPR30). GPER is a G-protein coupled receptor (GPCR) structurally unrelated to nuclear estrogen receptors and lacking DNA binding sites (5, 6). Accumulated evidence suggests that GPER is a membrane receptor for 17*β*-estradiol (7, 8). In 2005, two studies from different groups demonstrated specific binding of 17*β*-estradiol to GPER in GPER-transfected COS7 and HEK293 cells as well as several breast cancer cell lines (2, 3). Activation of GPER by 17*β*-estradiol leads to rapid activation of G proteins (2, 6, 9) and cell membrane-associated secondary signaling such as generation of cAMP, EGFR-signaling, and expression of Bcl-2, nerve growth factor, and cyclin D (10–12). Extensive studies have suggested the involvement of GPER in numerous estrogen-mediated physiological functions in mammals (8), including lipid metabolism and vascular function (13), insulin secretion (14), growth plate fusion (15), and reproductive functions such as primordial follicle formation in ovaries (16), as well as regulation of vitellogenesis (17) and oocyte meiotic arrest in fishes (7). GPER mRNA is widely expressed in mammalian reproductive tissues such as mammary glands, ovaries, oviducts, testes, and prostate glands (8, 16, 18–21). For example, GPER mRNA and protein are expressed in the thecal and granulosa cells of hamster ovaries (18) where GPER mediates the 17*β*-estradiol-stimulated primordial follicle formation in these cells (16). GPER has been implicated in estrogen-induced firing activity of calcium oscillations in luteinizing hormone releasing hormone (LHRH) neurons, in uterine proliferation and estrogen-induced augmentation of the oxytocin uterine contraction response, in the growth and proliferation of endometrial cells, apoptosis during spermatogenesis, and in breast, uterine, endometrial, and ovarian and testicular tumorigenesis (8, 12, 20, 22–24). However, whether this estrogen receptor plays a role in mammalian reproduction is still controversial because a few GPER knockout studies conducted in mice have suggested that GPER is not critical for mammalian reproduction due to lack of an effect on fertility and oocyte development (14, 25–28).

In the present study, *gper^−/−^* mutant zebrafish were generated, and fertility was observed over an extended period in order to comprehensively investigate the role of Gper in reproduction. Interestingly, we found a significant accumulation of early vitellogenic follicles (Stage III ovarian follicles) and fewer later stage follicles, indicating growth arrest and a reduction in fertility in the *gper^−/−^* females. This ovarian follicle growth tardiness was accompanied by reduced circulating levels of vitellogenin and by lower RNA and protein levels of epidermal growth factor receptor (Egfr). Our results suggest that gper has roles in ovarian development and female fertility in zebrafish.

## Materials and Methods

### Animals

Sanger AB Tübingen strain (SAT) of zebrafish (*Danio rerio*) used here originated from the Zebrafish International Resource Center and then propagated in our lab following previously published guidelines (29). The investigations have been approved by the Institutional Animal Care and Use Committee (IACUC) at East Carolina University.

### TALEN Assembly and *In Vitro* Synthesis of TALEN mRNAs

**Figure 1:**
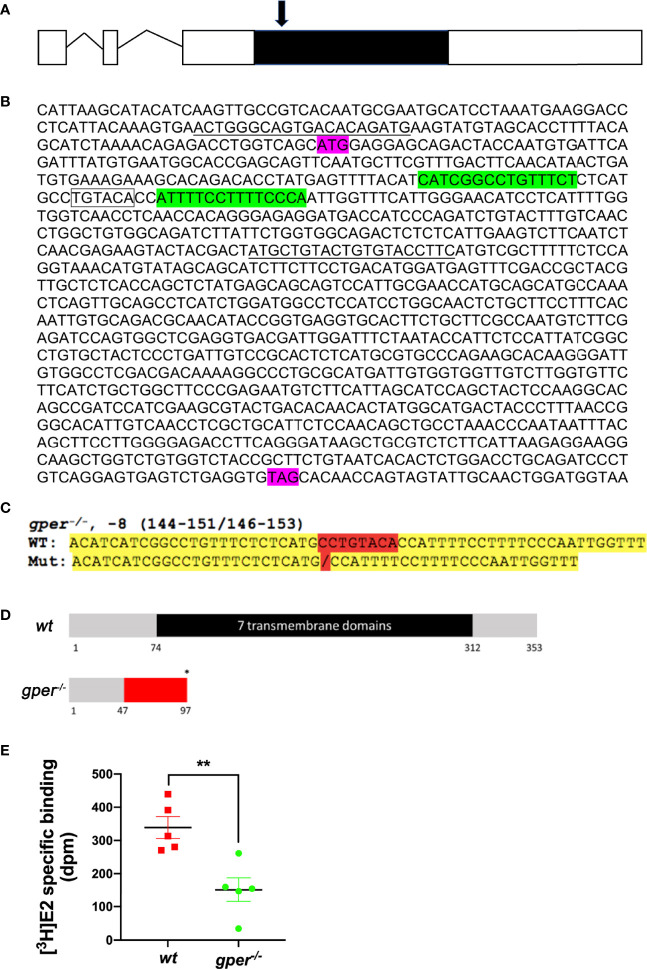
Targeted genetic modification of *gper* and decreased estradiol binding in zebrafish. **(A)** Gene structure for the *gper* gene. Zebrafish gper contains three exons. Line indicates intron, open boxes indicate uncoded 5′ or 3′ region, while a filled black box indicates an entire coding region of *gper*. Arrow indicates approximate position of TALEN targeting region. **(B)** Showing TALEN targeting sites that are highlighted in green in *gper* cDNA. Translation start site (ATG) and stop codon (TAG) are indicated in purple. A restriction enzyme recognition site (BsrGI: TGTACA) is indicated by the box. Forward and reverse PCR primers for amplification of the targeted region including TALEN targeting sites are underlined. **(C)** Verified sequence change in *gper^−/−^*. Mutated nucleotides highlighted in red and yellow indicate flanking sequences. **(D)** Predicted truncated protein in *gper^−/−^* (red indicates missense amino acids, an asterisk indicates a premature stop codon). Numbers under the box indicate the amino acid number from the translation start site. **(E)** Lower estradiol binding was found in *gper^−/−^* ovarian membrane compared with wildtype (*wt*). n = 5. **p < 0.01.

Target site and assembled TALEN molecules were designed and synthesized according to a previous method (30). We identified the target site sequence in exon 3 which is the only protein-coding exon of the *gper* and satisfies the requirements for TALEN target design (**Figure 1**). The target was selected near the beginning of the coding sequence (forward target site sequence: CATCGGCCTGTTTCT, reverse target site sequence: TGGGAAAAGGAAAAT, and spacer sequence with a BsrGI restriction enzyme site: CTCATGCCTGTACACC). All assembled TALEN vectors were confirmed using Sanger sequencing. The assembled TALEN vectors were linearized with Not I, gel extracted, and purified using the QIAquick gel extraction kit according to the manufacturer’s specifications (Qiagen, MD), and mRNAs transcribed using SP6 mMACHINE kit (Ambion, TX). The transcribed mRNAs were stored at −80°C until use. Immediately prior to microinjection, mRNA was diluted into workable concentrations (100 ng/µl) with nuclease-free water and mixed with an equal volume of 0.5% phenol red solution (Sigma P0290).

### Generation of *gper^−/−^* Mutant Line

Approximately 1 nl of TALEN transcripts (100 ng/µl) was microinjected into one cell stage embryos within 30 min of spawning. Microinjection was driven by compressed N2 gas under control of PV820 Pneumatic PicoPump (World Precision 13 Instruments, Sarasota, FL), using a microcapillary pipette attached to a micromanipulator, under a Leica MZ6 microscope (Leica, Germany). To estimate mutagenesis efficiency, embryos without microinjection were designated as wildtype (*wt*) and used as the control group. Genomic DNA was extracted from 30 normally developing *wt* or TALENs-transcripts-microinjected embryos two-days post fertilization (dpf), using the HotSHOT method (31). Mutation rates were estimated by comparing the band intensities of undigested PCR products, to the intensities of digested PCR products using restriction enzyme (BsrGI) digestion analysis (29).

To identify germline-transmitted mutations, the remaining F0 founder embryos were raised to adulthood and outcrossed with *wt* fish. Genomic DNA from each cross was extracted from 30 randomly selected and pooled F1 embryos, and the status of the target site was analyzed *via* PCR amplification, restriction enzyme digestion analysis, and DNA sequencing as described above. The remaining F1 embryos with identified frameshift mutations were raised to adulthood and individually genotyped. Genomic DNA was extracted from part of the caudal fin of each adult fish in 50 μl hot alkaline solution, then analyzed as stated above. Heterozygous F1 adults that carried the same frameshift mutant alleles were then crossed with each other. These crosses yielded *wt*, heterozygous, and homozygous F2 fish that were further genetically and physiologically characterized.

### Membrane Receptor Binding Assay

Membrane E2 binding was conducted as described previously (32) using [^3^H]-17*β*-estradiol ([^3^H]-E2) (91.3 Ci/mmol, American Radiolabeled Chemicals, St. Louis, MO). Plasma membrane fractions of ovaries were obtained following established procedures with few modifications (33). The ovaries from *wt* or *gper^−/−^* fish were washed with assay buffer and then sonicated for 6 s, followed by a 1,000 g centrifugation for 7 min to remove any nuclear and heavy mitochondrial material. The resulting supernatant was centrifuged at 20,000 g for 20 min to obtain the plasma membrane fraction. Estrogen receptor binding in the membrane fractions was measured in a single-point assay as described previously (32). One set of tubes contained radiolabeled E2 alone (final concentration: 4.0–4.3 nM, total binding), while another set also contained cold E2 competitor at a 1,000-fold greater concentration (4 µM) to measure non-specific binding. After a 30-min incubation at 4°C with the membrane fractions (160–240 µg membrane protein), the reaction was stopped by filtration (Whatman GF/B filters, presoaked in assay buffer; Brandel Inc., Gaithersburg, MD, Fisher Scientific). The filters were washed twice with 25 ml assay buffer, and bound radioactivity was measured by scintillation counting. The results were expressed as specific binding of [^3^H]-E2.

### Histology

Three females from each genotype were collected at 8 am (1 h before lights turned on) and deeply anesthetized in a lethal dose of MS-222 (300 mg/L buffered solution) for 1 min. Fresh ovaries were fixed overnight in 10% buffered formalin (Fisher Scientific), washed in tap water, dehydrated through increased concentrations of ethanol (70, 80, 90, 100%, 30 min each) and embedded in JB-4 resin (Polysciences, Warrington, PA). Sections of 5 μm were cut and stained with hematoxylin and eosin.

### Spawning and Fertility

After all zebrafish lines reached maturity at ~4-months of age, at least 10 homozygous mutant female fish were crossed with *wt* fertility-confirmed males. Production of the offspring for each genotype was recorded daily for a period of two weeks, following a two-week acclimation period. The paired fish were fed with a commercial food (Otohime B2, Reed Mariculture, CA, USA) and supplemented with newly hatched brine shrimp *Artemia* (Brine Shrimp Direct, Utah, USA) twice a day. Fish were only considered acclimated and suitable for fertility testing if they previously spawned at least five times. In the following 2-week span, the numbers of spawned embryos were manually counted daily. Spawning frequency is defined as the number of times a female produced fertilized embryos in a two-week examination period.

### Follicle Isolation and Quantification

Zebrafish are photoperiodic breeders whose reproductive cycles are controlled by light cycles. At 9 am the lights turn on in the fish lab, prompting the fish to begin spawning. Typically, oocyte maturation occurs around 6 am; oocyte ovulation occurs around 8 am; and spawning occurs between 9 and 11 am. Sampling ovaries at the onset of spawning (9:30 am) allows us to examine the number of oocytes that failed to reach ovulation. The follicles (n = 8 female) were isolated according to our previous research (34). The follicles were divided into five different stages based on follicular size and morphological criteria (35) with a slight modification (34). Since Stage III covers a wide range of follicles, we further divided Stage III into four subgroups: 340–400, 400–450, 450–550, and 550–690 µm diameter oocytes.

### Vitellogenin Quantification

Blood was collected from dorsal aorta of zebrafish and stored at −80°C. Blood vitellogenin concentration was quantified using an enzyme-linked immunosorbent assay (ELISA) kit following the manufacturer’s protocol (Caymen Chemical Company, Ann Arbor, MI; Cat: 10004995). Blood samples were diluted 10^−5^, and vitellogenin was normalized to total blood protein concentration as determined by the Bradford method using bovine serum albumin as the standard reference protein.

### Western Blotting

Expression of Egfr in Stage III (400–450 µm) follicles was confirmed by Western blot analysis using a commercial antibody against zebrafish Egfr (# 55473, AnaSpec, Fremont, CA; 1:250) which has been previously characterized (36). Image analyzing software (ImageJ) was used to estimate relative densitometries of EGFR bands which were then normalized to the expression of *α*-tubulin (37).

### Quantitative PCR

At 5:30 am, brain, liver and gonads were dissected from 4-month old female zebrafish and immediately placed in 1.7 ml RNase-free microcentrifuge tubes (GeneMate) on ice, containing RNAzol (Molecular Research Center, Inc., OH. Catalog: RN 190). Total RNA was extracted using RNAzol and a Qiagen RNeasy kit according to the manufacturer’s protocol. Reverse transcription was performed using SuperScript III Reverse Transcriptase following the manufacturer’s instructions (Invitrogen, Carlsbad, CA).

Primers used for qPCR were described previously (38). qPCR was performed using the SYBR green (Invitrogen) with C1000 Touch Thermal Cycler (Bio-Rad). The protocol consisted of a cycling profile of 30 s at 95°C, 30 s at 58°C, and 45 s at 72°C for 45 cycles followed by a melting curve test. House-keeping-gene *gapdh* was used as control.

### Untargeted Metabolomic Analysis and Measurement of Estrogen Levels in the Ovaries

Ovaries were collected from 4-month old healthy and mature *wt* or *gper^−/−^* at 5:30 am prior to oocyte maturation. The ovaries were immediately sonicated (Sonic Dismembrator, Fisher Scientific) in 600 µl of optima grade water (Fisher Scientific, Fairlawn, NJ) on ice. Steroids were extracted from the samples using a liquid–liquid extraction (LLE) method. Briefly, 2.4 ml methanol:water in 1:1 ratio containing 0.1% formic acid was added to each sample and vortexed for 5 min. The sample was centrifuged for 15 min at 14,000 g, and the supernatant was transferred into a 16 × 125 mm borosilicate glass tube (VWR, Radnor, PA). LLE was conducted using 3 ml methyl-tert-butylether (MTBE) (Fisher Scientific) added to the supernatant. The mixture was vortexed for 15 min and centrifuged for 2 min at 800 g. The organic phase was collected, and the extraction process was repeated two more times with the remaining aqueous phase. The extracted organic fractions were combined in the same tube and dried under nitrogen at a room temperature. Each sample was resuspended in 100 µl of sample buffer (70 acetonitrile:30 water containing 0.1% formic acid) for liquid chromatography/mass spectrometry (LCMS) analysis.

To quantify estrone and 17*β*-estradiol levels in the samples, external standard calibration curves were established using six different concentrations of these estrogens (0.005, 0.01, 0.05, 0.1, 0.5, 1 μg/ml) (Sigma-Aldrich, St. Louis, Missouri). Extracted steroids were identified and quantified using an Eskigent 425 microLC/SCIEX 5600 + Triple time-offlight mass spectrometer. Samples and standards in autosampler vials were loaded in a refrigerated holder (4°C). A HALO C18, 2.7 µm, 0.5 × 50 mm microLC column purchased from Eksigent (Dublin, CA) was maintained at 25°C. The flow rate was 10 µl/min, and 5 µl of sample was injected. A standard 30 min gradient was used with mobile phase A: water with 0.1% formic acid and mobile phase B: acetonitrile. For data quantification, the integration of peak areas was conducted using PeakView and MultiQuant (SCIEX), and an external standard calibration curve was used to calculate the hormone amount and normalized by the ovary weight. Independent data acquisition was utilized to collect the top 20 MS/MS. Metabolomics analysis was conducted using MarkerView (SCIEX, Framingham, MA) and PeakView (SCIEX).

### Statistical Analysis

All results were analyzed using GraphPad Prism 7.0a (San Diego, CA, US) and presented as mean ± SEM. Significant differences among paired treatment groups were determined using Student’s t-test. Statistical significance was set at p <0.05.

## Results

### Generation of *gper^−/−^* Mutant Line

The protein-coding sequence of Gper is located exclusively in the third exon on chromosome 3 of zebrafish (**Figure 1A**). Therefore, we targeted the third exon of gper (**Figure 1B**). In *gper^−/−^* mutant line (*gper*^ecu18/ecu18^), an eight-nucleotide deletion mutation was generated in exon 3 (**Figure 1C**) that caused a frameshift resulting in truncation of the transcript prior to the region encoding the first transmembrane domain of *gper* (**Figure 1D**). It is predicted that the subsequent transmembrane domains were not synthesized (**Figure 1D**). F0 mosaics were raised to the adult stage and crossed with *wt* fish to generate the heterozygous F1 generation. The genotyped heterozygous F1 generation would then inter-crossed to generate F2 homozygous *gper^−/−^* mutants. Moreover, estradiol binding to ovarian membranes of *gper^−/−^* mutants was significantly lower than that of *wt* ovarian plasma membranes (**Figure 1E**).

### Reduced Fertility in *gper^−/−^* Female Fish

**Figure 2:**
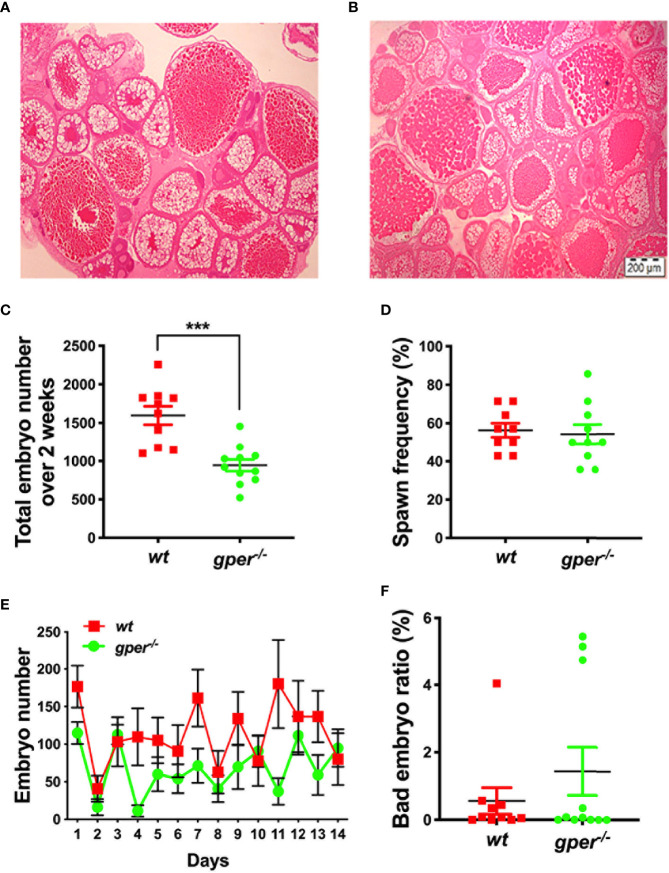
Reduced fertility in *gper^−/−^* female zebrafish. Hematoxylin & Eosin staining of a representative section from wildtype (*wt*) ***(*A*)*** and *gper^−/−^* **(B)** ovaries (n = 3 for each genotype). Both representative pictures depict Stage I–Stage IV oocytes. **(C)** Total number of embryos per female produced by *gper^−/−^* female zebrafish individually paired with fertility confirmed *wt* males over a -two-week period following two-week acclimation was significantly less than those from *wt* pairs. **(D)** Averaged spawn frequency (%) in two weeks. **(E)** The number of embryos per female produced daily in *gper^−^*^/^*^−^* was less than those from *wt*. **(F)** No significant difference in the rate of inviable embryos obtained in *gper^−^*^/^*^−^* compared to those in *wt*. n = 10 pairs for *wt*, n = 11 pairs for *gper^−^*^/^*^−^*. ***p < 0.001. Scale bar: 200µm.

No obvious morphological differences between ovaries of mutant females and *wt* were observed. Both the *gper^−/−^* and *wt* appeared to contain comparable numbers of different stages of oocytes (**Figures 2A, B**). Interestingly, we observed a 40.85% reduction in the total number of embryos produced by the *gper^−/−^* mutants compared to *wt* over a 2-week span (**Figure 2C**). However, the spawning frequency was similar in *gper^−/−^* in comparison to *wt* (**Figure 2D**). Additionally, a significant daily reduction in embryo numbers was observed in *gper^-/-^* compared to wt (**Figure 2E**). Lastly, we found no statistically significant difference in the ratio of abnormal embryos in the mutants compared to the *wt* fish (**Figure 2F**).

### Accumulated Early Vitellogenic Follicles and Reduced Plasma Vitellogenin Levels in *gper^−/−^* Females

**Figure 3:**
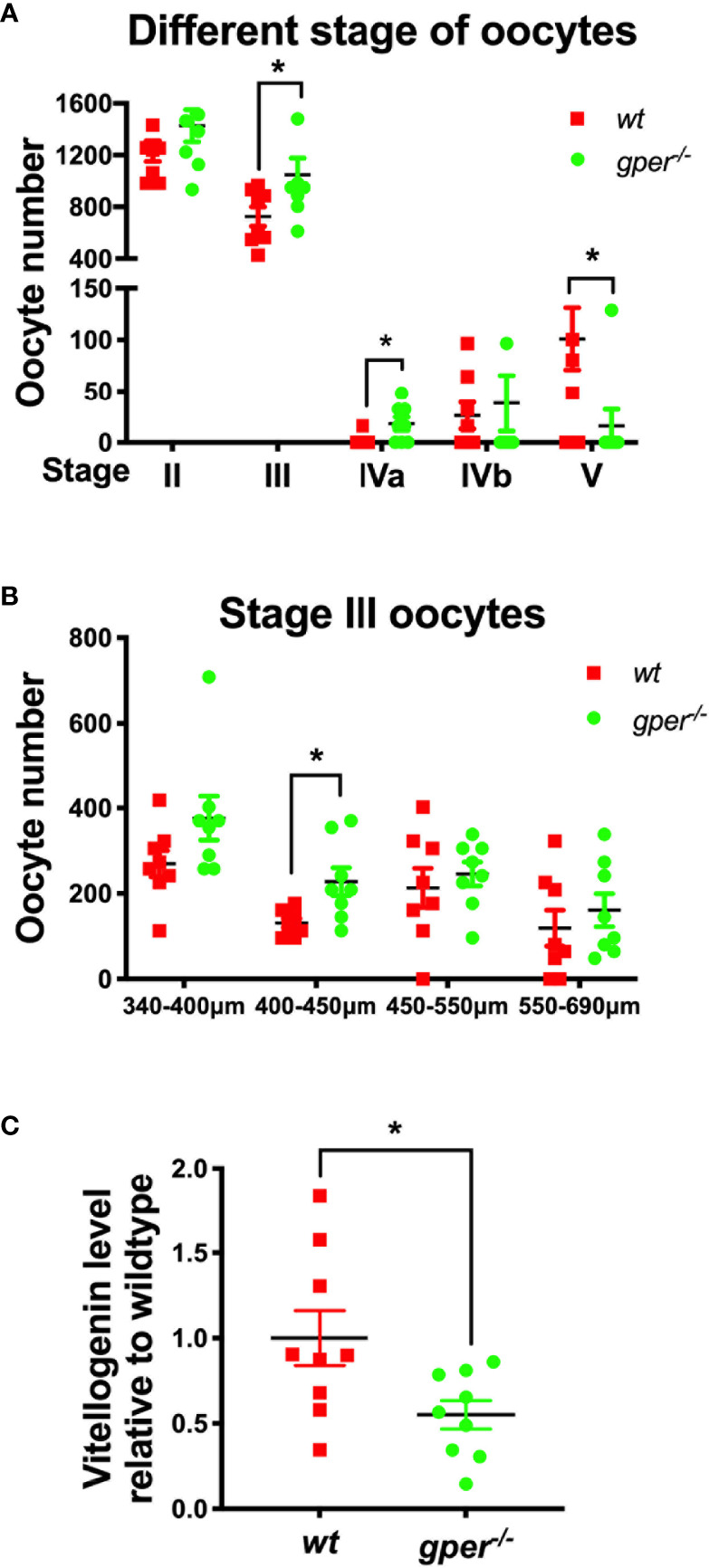
Accumulation of early vitellogenic Stage III oocytes and low plasma vitellogenin level in *gper^−^*^/^*^−^* female zebrafish. **(A)** More Stage III and Stage IVa, while less Stage V oocytes, were found in *gper^−/−^* (n = 8) ovaries than those in *wt* (n = 8). **(B)** More Stage III oocytes with diameter range from 400 to 450 µm were found in *gper^−/−^* ovaries (n = 8). **(C)** Decrease of plasma vitellogenin levels were observed in *gper^−/−^* zebrafish females with fertility confirmed prior to sampling (n = 9). *p < 0.05.

Interestingly, the number of Stage V oocytes (ovulated) was significantly lower in *gper^−/−^* mutants compared to *wt* (**Figure 3A**), which agreed with the subfertility phenotype found in *gper^−/−^* females (**Figure 2C**). We observed no difference between the number of Stage II follicles (pre-vitellogenesis) in *gper^−/−^* compared to those in *wt* (**Figure 3A**). But there was a significantly higher number of Stage III and IVa follicles in the *gper^−/−^* mutants than in the *wt* (**Figure 3A**), indicating possible dysfunction during vitellogenesis and oocyte maturation in *gper^−/−^* mutants. Since Stage III covers a wide range of different follicle sizes, we further divided Stage III into four groups. Specifically, *gper^−/−^* ovaries contained more early vitellogenic follicles (*i.e.*, more 400–450 µm follicles) than those in *wt* ovaries (**Figure 3B**). Furthermore, the concentration of vitellogenin in the blood of *gper^−/−^* females was significantly lower (~57% of control; p = 0.025) than that of *wt* fish (**Figure 3C**), indicating a vitellogenesis dysfunction in *gper^−/−^* females.

### Higher Estrogen but Reduced Egfr Expression in *gper^−/−^* Ovaries

**Figure 4:**
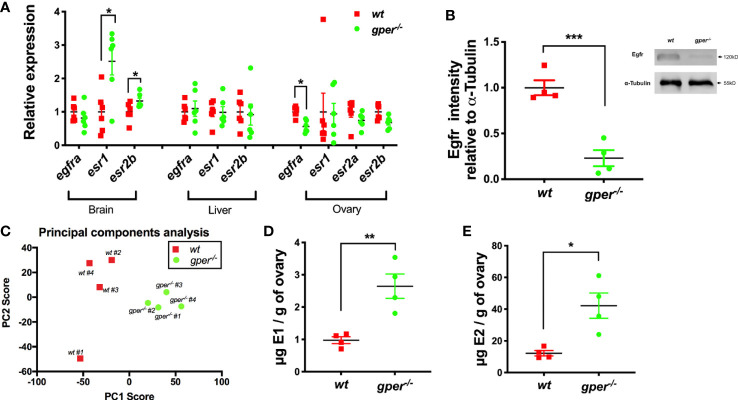
Higher estrogen levels and lower Egfra in *gper^−/−^* than *wt*. **(A)** Changes of *egfra* and *esr* in the brain, liver, and ovary in *gper^−^*^/^*^−^* female zebrafish. Expression levels were normalized relative to housekeeping gene *gapdhs* (n = 6). (*egfra*, epidermal growth factor receptor a; *esr*, estrogen receptor). **(B)** Reduced Egfr protein in Stage III follicles (400–500 µm) of *gper^−/−^* (n = 4). Insert, a representative Western blot of Egfr protein. **(C)** Distinct cluster separation of *gper^−^*^/^*^−^* away from *wt* shown by a PCA plot using data from untargeted metabolomics analyses. **(D–E)** Increase of estrone (E1) and 17*β*-estradiol (E2) were found in *gper^−/−^* ovaries in comparison to those from *wt*. The hormone values were determined using LC-MS (n = 4). *p < 0.05, **p < 0.01, ***p < 0.001.

17*β*-estradiol actions through its nuclear receptors are the most potent stimulators for vitellogenin synthesis (17, 39, 40). To further understand the role Gper may play in the reproductive system, we determined the levels of estrogens, and the expression of nuclear estrogen receptors, and Egfra in zebrafish tissues. In the brain, *esr1* and *esr2b* showed significant upregulation in *gper^−/−^*, possibly compensating for reduced estrogen signaling (**Figure 4A**). Mutant fish exhibited downregulation of *egfra* in ovaries but not in brains or livers (**Figure 4A**). In livers and ovaries, levels of *esr1*, *esr2a*, and *esr2b* were similar in *gper^−/−^* as in *wt* fish (**Figure 4A**). Furthermore, we found reduced expression of the Egfr protein in early vitellogenic follicles (*i.e.*, Stage III follicles, 400–450 µm) collected from *gper^−/−^* (**Figures 4B**).

To further investigate the role Gper plays in female reproduction, we conducted untargeted and targeted metabolomics analysis from *wt* and *gper^−/−^* ovaries and analyzed molecular profiles using LC-MS. For the untargeted analysis, we conducted principal component analysis (PCA) to view sample groupings. PCA scores plot (PC1, 46.4%; PC2, 16.4%) indicated there were distinct cluster differences between *wt* ovaries and *gper^−/−^* ovaries (**Figure 4C**). There were 8,755 metabolite features detected from the untargeted analysis where 22 features were significantly upregulated in the *gper^−/−^* with a p-value <0.01 and five-fold change and 119 features downregulated in the *gper^−/−^* with a p-value <0.01 and fivefold change when compared to the *wt* ovaries (data not shown). For the targeted analysis we included two estrogens, estrone (E1) and 17*β*-estradiol (E2). Significantly higher estrone (**Figure 4D**) and 17*β*-estradiol (**Figure 4E**) levels were detected in *gper^−/−^* ovaries compared to the *wt*.

## Discussion

The present results in zebrafish provide new evidence that GPER has a role in fertility *via* regulation of vitellogenesis and Egfr in female zebrafish. The nuclear estrogen receptors, ER*α* and ER*β*, have high binding affinities for estrogens and have long been known to have essential roles in estrogen regulation of reproductive and non-reproductive functions in male and female vertebrates. There is now clear evidence that GPER also binds estrogens such as 17*β*-estradiol specifically with relatively high affinity (Kd 2.7–3 nM) (2, 3, 27) to mediate a wide variety of estrogen actions in different cell and animal models (8, 13, 14). However, controversy still surrounds some of GPERs proposed functions for which conflicting results have been obtained so that GPER’s physiological role *in vivo* remains unclear (41, 42). Several diverse phenotypes with only partial overlap have been reported in different GPER mutant mouse lines, and none of them showed evidence of reproductive impairment (13, 14, 26, 43). Our main finding from the *gper^−/−^* lines is that female zebrafish are subfertile with a significant reduction of ovulated oocytes and a significant accumulation of early vitellogenic follicles (*i.e.*, Stage III ovarian follicles), indicating retarded follicle development beyond this stage. Moreover, plasma vitellogenin levels were significantly decreased in *gper^−^*^/^*^−^* females, and the expression of Egfr, which mediates EGF inhibition of ovarian follicle apoptosis (44) and is a regulator of ovarian steroid production in teleosts (45), was significantly reduced in Stage III follicles, suggesting plausible mechanisms for the decline in follicle growth in the *gper^−/−^* mutants. Collectively, these results demonstrate disruption of ovarian functions in zebrafish *gper^−/−^* mutants, and indicate an essential role for Gper in female reproduction in a vertebrate model. In light of these findings, as well as previous evidence for broad expression of GPER in the mammalian ovary and other reproductive tissues, the possible involvement of GPER in estrogen regulation of female reproduction needs to be re-examined in mammals. In the present study, Gper knockout male fish were fertile. We did not observe obvious reproductive phenotypes in *gper^−/−^* male zebrafish. Both Gper transcripts and proteins have been localized in testis and Leydig cells in mammals (46, 47). However, the functions of Gper in male vertebrate still needs to be defined.

### Low Egfr in Oocytes in *gper^−/−^* Fish

EGFR, also known as HER1 or erbB1, is a dimeric cell surface receptor that binds EGF, stimulating cells to proliferate. Lower expression levels of Egfr in *gper^−/−^* Stage III oocytes are consistent with previous observations indicating cross-talk between Gper and EGFR (10, 11, 36, 48). Estrogen stimulation transactivates EGFR *via* the action of matrix metalloproteinases and antisense oligonucleotide-mediated depletion of GPER inhibited EGFR transactivation by estrogen (49). The mechanism of how Gper affects EGFR expression is still unclear, but GPER may alter gene expression in response to estrogen (50). In MCF-7 cells, manipulation of GPER levels resulted in corresponding changes of MAPK activity (51) and progestin-induced growth inhibition (52). GPER also participates in the transcriptional activation of Bcl-2 in keratinocytes (53–55). Furthermore, the transcriptional activation of proto-oncogene c-fos stimulated by estrogen and phytoestrogens was through the activation of ERK1/2 by GPER in breast cancer cells (56). This may indicate Gper can regulate transcriptional activity in an indirect way. Secondly, estrogen activates GPER could promote second messengers and activate EGFR (10). Estradiol is conducive to the transcription of epidermal growth factor (EGF)-like factors through GPER in cumulus cells because this process can be inhibited by specific GPER inhibitor G15 (57). *Vice versa*, EGF also can activate the EGFR–ERK1/2 transduction pathway that stimulates the upregulation of GPER in nuclear estrogen receptor-negative breast cancer cells (58).

### Dysfunction During Early Vitellogentic Follicles (Stage III Ovarian Follicle Development)

Estrogens are pleiotropic hormones that regulate the growth and differentiation of many tissues, including ovarian follicles. In Stage III follicles in zebrafish, oocytes continue to grow predominantly due to the accumulation by endocytosis of a female-specific yolk precursor protein vitellogenin, which is synthesized in the liver in response to 17*β*-estradiol secreted from the ovarian follicle cells (35). We observed underdevelopment and buildup of Stage III oocytes in our *gper^−/−^*. This accumulation of Stage III oocytes is consistent with the peak of *gper* expression occurring during Stage III of oocyte development (32).

The accumulation of oocytes during Stage III also reflects a dysfunction with vitellogenin synthesis as indicated by the decrease in circulating vitellogenin levels, with Gper playing an important but not essential role (17). Chen et al. showed that the inhibition of Gper results in the significant attenuation of the E2 induction of vitellogenin production (17). In addition, lower EGFR level may also contributed to vitellogenin synthesis problem in *gper^−/−^* female zebrafish. EGFR can stimulate ovarian development in the mud crab *Scylla paramamosain* (59). hEGF treatment of ovary explants from late vitellogenic stage crabs significantly increased vitellogenin receptor (*VgR*), and this hEGF-induced effect could be suppressed by pretreatment with EGFR inhibitor AG1478 and PD153035 (59). Less vitellogenin synthesis caused by a reduction in Gper function, and less Egfr could likely result in inhibition of oocyte growth, which may cause the subfertility found in our *gper^−/−^* females.

In the present study, ovarian estrogen levels are increased possibly due to feedback of lack of Gper signals. Nuclear estrogen receptors seem to be unchanged at least at transcript levels in *gper^−/−^* in the liver. It is possible that estrogen may signal *via* Gper to regulate translation of estrogen receptors to affect vitellogenesis in the liver. Alternatively, non-genomic Gper signals may affect translation and translation of vitellogenin directly (not *via* classical estrogen receptor). Whether Gper has direct or indirect effects in the regulation of vitellogenin and its underlying mechanism needs further investigation.

### Effect of Gper on the Reproduction

In contrast to our findings, using CRISPR/Cas9, Crowder et al. found that zebrafish *gper^−/−^* mutants had no effect on fertility, sex determination, or ovary development (60). The reasons for their negative findings are unclear, but it should be noted that they followed a different fertility protocol to that used in the present study. The spawning trials were conducted with 10-month old fish held under group housing conditions and bred once every 2 weeks in the Crowder et al. study. However, in the present study, spawning trials were conducted with younger fully mature fish (4 months old), housed in pairs, and fed to satiation daily, and checked for spawning and the production of embryos every day over a 2-week period, resulting in a more accurate collection of fertility data over time. Before actual counting, we also gave the fish an acclimation period to ensure the fish were healthy, well fed, and had a previous history of successful spawning. Our *gper^−/−^* females also produced similar number of embryos compared to *wt* at some time points (**Figure 2E**). Therefore, breeding the fish only once every 2 weeks may not detect differences in fertility. Reproductive success of zebrafish in the laboratory has previously shown to be highly variable, cyclical, decreased as the colony size increases, and intermittent, demonstrating the need for large numbers of spawning trials to obtain accurate estimates of reproductive output (61). Second, several other factors, such as the targeting strategy, the genetic background, the breeding strategy, nutrition, and environmental components can severely affect the observed phenotype (62). The strain of zebrafish used in our lab comes from AB Tubingen compared to their AB zebrafish strain. It is possible that compensation had occurred by heritable changes that mitigate for the loss of Gper in the pure AB strain used by Crowder et al. Compensation has been demonstrated in the AB strain for the loss of several genes critical for the regulation of zebrafish reproduction, such as *gnrh3*, *kiss1r*, and *kiss2r* (63–65). Finally, this is the first time in any species that membrane estradiol binding was shown to be reduced in *gper^−/−^* ovaries.

Gper has previously been shown to mediate estrogen maintenance of meiotic arrest of full grown (Stage IVa) zebrafish oocytes (32, 66). However, oocyte maturation is merely delayed a few hours, not inhibited, through this mechanism (32, 66). Therefore, the whole animal studies conducted here would most likely not detect loss of this regulatory mechanism in *gper^−^*^/^*^−^* fish. Comprehensive investigations of estrogen regulation of meiotic arrest of Stage IVa oocytes in *gper^−^*^/^*^−^* females are clearly warranted. In addition, the functions of Gper in earlier stage oocytes should be investigated in this model. For example, estrogens have been shown to maintain meiotic arrest in late Stage III oocytes (500–550μm diameter) which have higher expression of *gper* mRNA than Stage IVa oocytes (32).

Overall, we have demonstrated the role of gper in oocyte development and fertility. Together these results indicate that Gper has a role in female reproduction in zebrafish.

## Data Availability Statement

The raw data supporting the conclusions of this article will be made available by the authors, without undue reservation.

## Ethics Statement

The animal study was reviewed and approved by Animal Care and Use Committee (IACUC) at East Carolina University.

## Author Contributions

YZ conceived the project and generated knockouts. X-JW, PT, and YZ designed the experiments, performed the experiments, and analyzed the data, and wrote the manuscript. MW conducted ovary histology, contributed to the draft and discussion. X-JW and KK did liquid–liquid extraction and LCMS. AC and PT conducted membrane E2 specific binding, measured blood vitellogenin levels and contributed to a revision of the manuscript and the discussion. All authors contributed to the article and approved the submitted version.

## Funding

This work was supported by the NIH GM100461 to YZ.

## Conflict of Interest

The authors declare that the research was conducted in the absence of any commercial or financial relationships that could be construed as a potential conflict of interest.

## References

[1] Vrtačnik P Ostanek B Mencej-Bedrač S Marc J. The many faces of estrogen signaling. Biochem Med: Biochem Med (2014) 24:329–42. 10.11613/BM.2014.035 PMC4210253 25351351

[2] Thomas P Pang Y Filardo E Dong J. Identity of an estrogen membrane receptor coupled to a G protein in human breast cancer cells. Endocrinology (2005) 146:624–32. 10.1210/en.2004-1064 15539556

[3] Revankar CM Cimino DF Sklar LA Arterburn JB Prossnitz ER. A transmembrane intracellular estrogen receptor mediates rapid cell signaling. Science (2005) 307:1625–30. 10.1126/science.1106943 15705806

[4] Lösel R Wehling M. Nongenomic actions of steroid hormones. Nat Rev Mol Cell Biol (2003) 4:46. 10.1038/nrm1009 12511868

[5] Carmeci C Thompson DA Ring HZ Francke U Weigel RJ. Identification of a gene (GPR30) with homology to the G-protein-coupled receptor superfamily associated with estrogen receptor expression in breast cancer. Genomics (1997) 45:607–17. 10.1006/geno.1997.4972 9367686

[6] Gaudet H Cheng S Christensen E Filardo E. The G-protein coupled estrogen receptor, GPER: The inside and inside-out story. Mol Cell Endocrinol (2015) 418:207–19. 10.1016/j.mce.2015.07.016 26190834

[7] Thomas P Alyea R Pang Y Peyton C Dong J Berg A. Conserved estrogen binding and signaling functions of the G protein-coupled estrogen receptor 1 (GPER) in mammals and fish. Steroids (2010) 75:595–602. 10.1016/j.steroids.2009.11.005 19931550 PMC2885585

[8] Prossnitz ER Barton M. The G-protein-coupled estrogen receptor GPER in health and disease. Nat Rev Endocrinol (2011) 7:715. 10.1038/nrendo.2011.122 21844907 PMC3474542

[9] Filardo E Quinn J Pang Y Graeber C Shaw S Dong J. Activation of the novel estrogen receptor G protein-coupled receptor 30 (GPR30) at the plasma membrane. Endocrinology (2007) 148:3236–45. 10.1210/en.2006-1605 17379646

[10] Filardo EJ Thomas P. GPR30: a seven-transmembrane-spanning estrogen receptor that triggers EGF release. Trends Endocrinol Metab (2005) 16:362–7. 10.1016/j.tem.2005.08.005 16125968

[11] Filardo EJ Quinn JA Frackelton AR Jr. Bland KI. Estrogen action via the G protein-coupled receptor, GPR30: stimulation of adenylyl cyclase and cAMP-mediated attenuation of the epidermal growth factor receptor-to-MAPK signaling axis. Mol Endocrinol (2002) 16:70–84. 10.1210/mend.16.1.0758 11773440

[12] Albanito L Madeo A Lappano R Vivacqua A Rago V Carpino A. G protein–coupled receptor 30 (GPR30) mediates gene expression changes and growth response to 17β-estradiol and selective GPR30 ligand G-1 in ovarian cancer cells. Cancer Res (2007) 67:1859–66. 10.1158/0008-5472.CAN-06-2909 17308128

[13] Haas E Bhattacharya I Brailoiu E Damjanovic M Brailoiu GC Gao X. Regulatory role of G protein–coupled estrogen receptor for vascular function and obesity. Circ Res (2009) 104:288–91. 10.1161/CIRCRESAHA.108.190892 PMC2782532 19179659

[14] Mårtensson UE Salehi SA Windahl S Gomez MF Swärd K Daszkiewicz-Nilsson J. Deletion of the G protein-coupled receptor 30 impairs glucose tolerance, reduces bone growth, increases blood pressure, and eliminates estradiol-stimulated insulin release in female mice. Endocrinology (2009) 150:687–98. 10.1210/en.2008-0623 18845638

[15] Windahl SH Andersson N Chagin AS Martensson UE Carlsten H Olde B. The role of the G protein-coupled receptor GPR30 in the effects of estrogen in ovariectomized mice. Am J Physiol-Endocrinol Metab (2009) 296:E490–6. 10.1152/ajpendo.90691.2008 19088255

[16] Wang C Prossnitz ER Roy SK. G protein-coupled receptor 30 expression is required for estrogen stimulation of primordial follicle formation in the hamster ovary. Endocrinology (2008) 149:4452–61. 10.1210/en.2008-0441 PMC2553386 18499747

[17] Chen Y Tang H He J Wu X Wang L Liu X. Interaction of nuclear ERs and GPER in vitellogenesis in zebrafish. J Steroid Biochem Mol Biol (2019) 189:10–8. 10.1016/j.jsbmb.2019.01.013 30711474

[18] Wang C Prossnitz ER Roy SK. Expression of G protein-coupled receptor 30 in the hamster ovary: differential regulation by gonadotropins and steroid hormones. Endocrinology (2007) 148:4853–64. 10.1210/en.2007-0727 17640985

[19] Li Y-R Ren C-E Zhang Q Li J-C Chian R-C. Expression of G protein estrogen receptor (GPER) on membrane of mouse oocytes during maturation. J Assisted Reprod Genet (2013) 30:227–32. 10.1007/s10815-013-9942-z PMC3585672 23420106

[20] Chimento A Sirianni R Casaburi I Pezzi V. GPER signaling in spermatogenesis and testicular tumors. Front Endocrinol (2014) 5:30. 10.3389/fendo.2014.00030 PMC3944538 24639669

[21] Popli P Sirohi VK Manohar M Shukla V Kaushal JB Gupta K. Regulation of cyclooxygenase-2 expression in rat oviductal epithelial cells: Evidence for involvement of GPR30/Src kinase-mediated EGFR signaling. J Steroid Biochem Mol Biol (2015) 154:130–41. 10.1016/j.jsbmb.2015.07.019 26241029

[22] Sirianni R Chimento A Ruggiero C De Luca A Lappano R Andò S. The novel estrogen receptor, G protein-coupled receptor 30, mediates the proliferative effects induced by 17β-estradiol on mouse spermatogonial GC-1 cell line. Endocrinology (2008) 149:5043–51. 10.1210/en.2007-1593 18566133

[23] Noel SD Keen KL Baumann DI Filardo EJ Terasawa E. Involvement of G protein-coupled receptor 30 (GPR30) in rapid action of estrogen in primate LHRH neurons. Mol Endocrinol (2009) 23:349–59. 10.1210/me.2008-0299 PMC2654512 19131510

[24] Maiti K Paul J Read M Chan E Riley S Nahar P. G-1-activated membrane estrogen receptors mediate increased contractility of the human myometrium. Endocrinology (2011) 152:2448–55. 10.1210/en.2010-0979 21427217

[25] Pedram A Razandi M Levin ER. Nature of functional estrogen receptors at the plasma membrane. Mol Endocrinol (2006) 20:1996–2009. 10.1210/me.2005-0525 16645038

[26] Otto C Fuchs I Kauselmann G Kern H Zevnik B Andreasen P. GPR30 does not mediate estrogenic responses in reproductive organs in mice. Biol Reprod (2009) 80:34–41. 10.1095/biolreprod.108.071175 18799753

[27] Otto C Rohde-Schulz B Schwarz G Fuchs I Klewer M Brittain D. G protein-coupled receptor 30 localizes to the endoplasmic reticulum and is not activated by estradiol. Endocrinology (2008) 149:4846–56. 10.1210/en.2008-0269 18566127

[28] Levin ER. G protein-coupled receptor 30: estrogen receptor or collaborator? Endocrinology (2009) 150:1563–5. 10.1210/en.2008-1759 PMC2659267 19307418

[29] Zhu Y Liu D Shaner ZC Chen S Hong W Stellwag EJ. Nuclear progestin receptor (pgr) knockouts in zebrafish demonstrate role for pgr in ovulation but not in rapid non-genomic steroid mediated meiosis resumption. Front Endocrinol (2015) 6:37. 10.3389/fendo.2015.00037 PMC4365747 25852646

[30] Huang P Xiao A Zhou M Zhu Z Lin S Zhang B. Heritable gene targeting in zebrafish using customized TALENs. Nat Biotechnol (2011) 29:699. 10.1038/nbt.1939 21822242

[31] Meeker ND Hutchinson SA Ho L Trede NS. Method for isolation of PCR-ready genomic DNA from zebrafish tissues. Biotechniques (2007) 43:610. 10.2144/000112619 18072590

[32] Pang Y Thomas P. Role of G protein-coupled estrogen receptor 1, GPER, in inhibition of oocyte maturation by endogenous estrogens in zebrafish. Dev Biol (2010) 342:194–206. 10.1016/j.ydbio.2010.03.027 20382141 PMC2874603

[33] Zhu Y Rice CD Pang Y Pace M Thomas P. Cloning, expression, and characterization of a membrane progestin receptor and evidence it is an intermediary in meiotic maturation of fish oocytes. Proc Natl Acad Sci (2003) 100:2231–6. 10.1073/pnas.0336132100 PMC151323 12574519

[34] Wu X-J Thomas P Zhu Y. Pgrmc1 knockout impairs oocyte maturation in zebrafish. Front Endocrinol (2018) 9:560. 10.3389/fendo.2018.00560 PMC6165893 30319543

[35] Selman K Wallace RA Sarka A Qi X. Stages of oocyte development in the zebrafish, *Brachydanio rerio*. J Morphol (1993) 218:203–24. 10.1002/jmor.1052180209 29865471

[36] Aizen J Thomas P. Role of Pgrmc1 in estrogen maintenance of meiotic arrest in zebrafish oocytes through Gper/Egfr. J Endocrinol (2015) 225:59–68. 10.1530/JOE-14-0576 25720537

[37] Schneider CA Rasband WS Eliceiri KW. NIH Image to ImageJ: 25 years of image analysis. Nat Methods (2012) 9:671. 10.1038/nmeth.2089 22930834 PMC5554542

[38] Wu X-J Williams MJ Patel PR Kew KA Zhu Y. Subfertility and reduced progestin synthesis in Pgrmc2 knockout zebrafish. Gen Comp Endocrinol (2019) 282:113218. 10.1016/j.ygcen.2019.113218 31301284 PMC6718323

[39] Norris DO Jones RE. Hormones and reproduction in fishes, amphibians, and reptiles. New York and London: Plenum Press (reprint New York, Springer; Softcover reprint of the original 1st ed. 1987 edition (2011).

[40] Griffin LB January KE Ho KW Cotter KA Callard GV. Morpholino-mediated knockdown of ERα, ERβa, and ERβb mRNAs in zebrafish (*Danio rerio*) embryos reveals differential regulation of estrogen-inducible genes. Endocrinology (2013) 154:4158–69. 10.1210/en.2013-1446 PMC3800766 23928376

[41] Olde B Leeb-Lundberg LF. GPR30/GPER1: searching for a role in estrogen physiology. Trends Endocrinol Metab (2009) 20:409–16. 10.1016/j.tem.2009.04.006 19734054

[42] Langer G Bader B Meoli L Isensee J Delbeck M Noppinger PR. A critical review of fundamental controversies in the field of GPR30 research. Steroids (2010) 75:603–10. 10.1016/j.steroids.2009.12.006 20034504

[43] Chunhe C Dehghani B Magrisso IJ Rick EA Bonhomme E Cody DB. GPR30 contributes to estrogen-induced thymic atrophy. Mol Endocrinol (2008) 22:636–48. 10.1210/me.2007-0359 PMC2262170 18063692

[44] Janz DM Van Der Kraak G. Suppression of apoptosis by gonadotropin, 17β-estradiol, and epidermal growth factor in rainbow trout preovulatory ovarian follicles. Gen Comp Endocrinol (1997) 105:186–93. 10.1006/gcen.1996.6820 9038251

[45] Mukherjee D Majumder S Moulik SR Pal P Gupta S Guha P. Membrane receptor cross talk in gonadotropin-, IGF-I-, and insulin-mediated steroidogenesis in fish ovary: an overview. Gen Comp Endocrinol (2017) 240:10–8. 10.1016/j.ygcen.2016.09.002 27616426

[46] Rago V Giordano F Brunelli E Zito D Aquila S Carpino A. Identification of G protein-coupled estrogen receptor in human and pig spermatozoa. J Anat (2014) 224:732–6. 10.1111/joa.12183 PMC4025899 24697543

[47] Kotula-Balak M Pawlicki P Milon A Tworzydlo W Sekula M Pacwa A. The role of G-protein-coupled membrane estrogen receptor in mouse Leydig cell function—in vivo and in vitro evaluation. Cell Tissue Res (2018) 374:389–412. 10.1007/s00441-018-2861-7 29876633 PMC6209072

[48] Filardo EJ. Epidermal growth factor receptor (EGFR) transactivation by estrogen via the G-protein-coupled receptor, GPR30: a novel signaling pathway with potential significance for breast cancer. J Steroid Biochem Mol Biol (2002) 80:231–8. 10.1016/S0960-0760(01)00190-X 11897506

[49] Sukocheva O Wadham C Holmes A Albanese N Verrier E Feng F. Estrogen transactivates EGFR via the sphingosine 1-phosphate receptor Edg-3: the role of sphingosine kinase-1. J Cell Biol (2006) 173:301–10. 10.1083/jcb.200506033 PMC2063820 16636149

[50] Prossnitz ER Arterburn JB Smith HO Oprea TI Sklar LA Hathaway HJ. Estrogen signaling through the transmembrane G protein–coupled receptor GPR30. Annu Rev Physiol (2008) 70:165–90. 10.1146/annurev.physiol.70.113006.100518 18271749

[51] Ahola TM Alkio N Manninen T Ylikomi T. Progestin and G protein-coupled receptor 30 inhibit mitogen-activated protein kinase activity in MCF-7 breast cancer cells. Endocrinology (2002) 143:4620–6. 10.1210/en.2002-220492 12446589

[52] Ahola TM Manninen T Alkio N Ylikomi T. G protein-coupled receptor 30 is critical for a progestin-induced growth inhibition in MCF-7 breast cancer cells. Endocrinology (2002) 143:3376–84. 10.1210/en.2001-211445 12193550

[53] Kanda N Watanabe S. 17β-estradiol inhibits oxidative stress-induced apoptosis in keratinocytes by promoting Bcl-2 expression. J Invest Dermatol (2003) 121:1500–9. 10.1111/j.1523-1747.2003.12617.x 14675202

[54] Kanda N Watanabe S. 17β-estradiol enhances the production of nerve growth factor in THP-1-derived macrophages or peripheral blood monocyte-derived macrophages. J Invest Dermatol (2003) 121:771–80. 10.1046/j.1523-1747.2003.12487.x 14632195

[55] Kanda N Watanabe S. 17β-estradiol stimulates the growth of human keratinocytes by inducing cyclin D2 expression. J Invest Dermatol (2004) 123:319–28. 10.1111/j.0022-202X.2004.12645.x 15245432

[56] Maggiolini M Vivacqua A Fasanella G Recchia AG Sisci D Pezzi V. The G protein-coupled receptor GPR30 mediates c-fos up-regulation by 17β-estradiol and phytoestrogens in breast cancer cells. J Biol Chem (2004) 279:27008–16. 10.1074/jbc.M403588200 15090535

[57] Zhang H Lu S Xu R Tang Y Liu J Li C. Mechanisms of estradiol-induced EGF-like factor expression and oocyte maturation via G protein-coupled estrogen receptor. Endocrinology (2020) 161:bqaa190. 10.1210/endocr/bqaa190 33068422

[58] Albanito L Sisci D Aquila S Brunelli E Vivacqua A Madeo A. Epidermal growth factor induces G protein-coupled receptor 30 expression in estrogen receptor-negative breast cancer cells. Endocrinology (2008) 149:3799–808. 10.1210/en.2008-0117 PMC2488235 18467441

[59] Lu B Jiang Q Liu A Huang H Ye H. Stimulatory roles of epidermal growth factor receptor (EGFR) in ovarian development of mud crab *Scylla paramamosain*. Gen Comp Endocrinol (2020) 299:113616. 10.1016/j.ygcen.2020.113616 32950581

[60] Crowder CM Romano SN Gorelick DA. G Protein–Coupled Estrogen Receptor Is Not Required for Sex Determination or Ovary Function in Zebrafish. Endocrinology (2018) 159:3515–23. 10.1210/en.2018-00685 PMC6137278 30169775

[61] Paull GC Van Look KJ Santos EM Filby AL Gray DM Nash JP. Variability in measures of reproductive success in laboratory-kept colonies of zebrafish and implications for studies addressing population-level effects of environmental chemicals. Aquat Toxicol (2008) 87:115–26. 10.1016/j.aquatox.2008.01.008 18308405

[62] Yoshiki A Moriwaki K. Mouse phenome research: implications of genetic background. ILAR J (2006) 47:94–102. 10.1093/ilar.47.2.94 16547366

[63] Spicer OS Wong TT Zmora N Zohar Y. Targeted Mutagenesis of the Hypophysiotropic Gnrh3 in Zebrafish (Danio rerio) Reveals No Effects on Reproductive Performance. PloS One (2016) 11:e0158141. 10.1371/journal.pone.0158141 27355207 PMC4927163

[64] Tang H Liu Y Luo D Ogawa S Yin Y Li S. The kiss/kissr systems are dispensable for zebrafish reproduction: Evidence from gene knockout studies. Endocrinology (2015) 156:589–99. 10.1210/en.2014-1204 PMC4298318 25406015

[65] Trudeau VL. Facing the Challenges of Neuropeptide Gene Knockouts: Why Do They Not Inhibit Reproduction in Adult Teleost Fish? Front Neurosci (2018) 12:302. 10.3389/fnins.2018.00302 29773976 PMC5943551

[66] Thomas P. Role of G-protein-coupled estrogen receptor (GPER/GPR30) in maintenance of meiotic arrest in fish oocytes. J Steroid Biochem Mol Biol (2017) 167:153–61. 10.1016/j.jsbmb.2016.12.005 28007532

